# Evaluation of the Effectiveness of Assistive Technology for Executive Function Support for People With Acquired Brain Injury: Protocol for Single-Case Experimental Designs

**DOI:** 10.2196/48503

**Published:** 2023-08-29

**Authors:** Em Bould, Robyn Tate, Grahame Simpson, Natasha Brusco, Lisa Licciardi, Libby Callaway

**Affiliations:** 1 Department of Occupational Therapy, Monash University Frankston Australia; 2 Kolling Institute, Faculty of Medicine and Health, The University of Sydney, Sydney Australia; John Walsh Centre for Rehabilitation Research, Northern Sydney Local Health District, Sydney Australia Sydney Australia; 3 Kolling Institute, Faculty of Medicine and Health, The University of Sydney, Sydney Australia; John Walsh Centre for Rehabilitation Research, Northern Sydney Local Health District, Sydney Australia; Brain Injury Rehabilitation Research Group, Ingham Inst Sydney Australia; 4 Rehabilitation, Ageing and Independent Living (RAIL) Research Centre, Monash University Frankston Australia; 5 Department of Occupational Therapy / Rehabilitation, Ageing and Independent Living (RAIL) Research Centre, Monash University Frankston Australia

**Keywords:** acquired brain injury, assistive technology, clinical research protocol, economic evaluation, executive function, single-case experimental design

## Abstract

**Background:**

Executive function, including prospective memory, initiating, planning, and sequencing everyday activities, is frequently affected by acquired brain injury (ABI). Executive dysfunction necessitates the use of compensatory cognitive strategies and, in more severe cases, human support over time. To compensate for the executive dysfunction experienced, growing options for electronic mainstream and assistive technologies may be used by people with ABI and their supporters.

**Objective:**

We outline the study protocol for a series of single-case experimental designs (SCEDs) to evaluate the effectiveness of smart home, mobile, and/or wearable technologies in reducing executive function difficulties following ABI.

**Methods:**

Up to 10 adults with ABI who experience executive dysfunction and have sufficient cognitive capacity to provide informed consent will be recruited across Victoria and New South Wales, Australia. Other key inclusion criteria are that they have substantial support needs for everyday living and reside in community dwellings. On the basis of the participant’s identified goal(s) and target behavior(s), a specific electronic assistive technology will be selected for application. Both identification of the target behavior(s) and selection of the assistive technology will be determined via consultation with each participant (and their key support person, if applicable). The choice of SCED will be individualized for each participant based on the type of technology used in the intervention, the difficulty level of the behavior targeted for change, and the anticipated rate of change. For each SCED, repeated measurements of the target behavior(s) during the baseline condition will provide performance data for comparison with the performance data collected during the intervention condition (with technology introduced). Secondary outcome measures will evaluate the impact of the intervention. The protocol includes 2 customizable Microsoft Excel spreadsheets for electronic record keeping.

**Results:**

Recruitment period is June 2022 through March 2024. Trial results for the individual participants will be graphed and analyzed separately using structured visual analysis supplemented with statistical analysis. Analysis will focus on important features of the data, including both within- and between-phase comparisons for response level, trend, variability, immediacy, consistency, and overlap. An exploratory economic evaluation will determine the impact on formal and informal support usage, together with quality of life, following the implementation of the new technological intervention.

**Conclusions:**

The study has been designed to test the cause-effect functional relationships between the intervention—in this case, electronic assistive technology—and its effect in changing the target behavior(s). The evaluation evidence gained will offer new insights into the application of various electronic assistive technologies for people who experience executive dysfunction following ABI. Furthermore, the results will help increase the capacity of key stakeholders to harness the potential of technology to build independence and reduce the cost of care for this population.

**Trial Registration:**

Australian New Zealand Clinical Trials Registry (ANZCTR) ACTRN12622000835741, https://www.anzctr.org.au/ACTRN12622000835741.aspx

**International Registered Report Identifier (IRRID):**

DERR1-10.2196/48503

## Introduction

### Overview

Acquired brain injury (ABI) refers to damage to the brain that occurs after birth and includes traumatic brain injury, stroke, infection, anoxia or hypoxia, and brain cancer to name the most common [1]. Cognitive impairments, including damage to higher-order executive functions, are frequently observed after ABI. Common executive function impairments include issues with prospective memory, flexibility, judgment, insight, planning and organization, and initiation of functional tasks [2]. Executive impairments can have a significant impact on functional outcomes and adversely affect participation in areas such as employment, leisure pursuits, relationships, and independent living [3-5].

### Assistive Technology for Executive Function Support

A growing range of electronic assistive technologies is available, which may compensate for a number of challenges to participation. For instance, smartphones and tablets used by the wider population are emerging as useful tools for developing independence and participation after ABI [6-9]. To date, most studies evaluating the efficacy of assistive technology in providing support to compensate for executive impairments have focused on prospective memory compared with other components of executive function. The strongest evidence to date has come from a small number of randomized controlled trials testing the efficacy of delivering reminders to reduce prospective memory failures through text messages or other auditory cues delivered by personal digital devices or mobile phones [10,11]. To date, few studies have investigated the efficacy of assistive technology in supporting other areas of executive function, such as supporting goal-directed behavior to undertake functional therapy [12,13] or enhancing executive monitoring of daily intentions [14].

As the assistive technology field has been rapidly evolving, there are growing platforms and means for delivering support for executive functions. For example, home hubs constitute a more specialized type of multifunctional device. One example of such devices is “Sofihub,” which provides home-based movement sensing, audio-prompting technology (similar in principle to “Alexa” or “Google Home”). Sofihub was designed and marketed for people with disabilities, including those with cognitive impairment [15]. Fernando et al [15] provided preliminary evidence of the effectiveness of Sofihub in augmenting human support for people with executive dysfunction, including impaired initiation. The system provided prerecorded verbal prompts to participants at set times or triggered by sensors detecting movement. The verbal prompts targeted self-care activities (eg, showering, teeth cleaning, and medication administration), household tasks (eg, cleaning and putting out garbage), and reminders to take necessary items (eg, phone, keys, and hearing aids) when leaving the house.

In Australia, the National Disability Insurance Agency has identified that assistive technology offers the potential to reduce the lifetime care costs of people with severe disabilities [16]. Nevertheless, to date, there is limited published evidence regarding the use of technology in the delivery of support to people with ABI in a range of community settings [17]. Specific to electronic personal assistive devices, there is insufficient evidence to recommend any practice standards [18]. This limits the capacity of people with ABI and their supporters, including family, carers, and health professionals, to effectively implement and harness emerging technologies within community living.

### Objectives

This paper outlines the study protocol for a series of single-case experimental designs (SCEDs) to evaluate the effectiveness of smart home, mobile, and/or wearable technologies for executive function support following ABI. The findings from the Sofihub trial [15] and other published evidence identify SCED as the principal methodology used to evaluate technological solutions for people with ABI [18]. The rigorous methodology of SCED enables it to test for cause-effect functional relationships between the intervention—in this case, smart home, mobile technology, or wearable technologies—and its effect in changing a behavior (eg, an increase in the number of steps in a specified task completed following a prompt from smart devices and a corresponding decrease in the number of verbal cues required from a support person) [19-21]. However, to date, many SCEDs have been of low scientific quality [22,23], and therefore, there is a need for more methodologically rigorous, evidence-based research to better understand the benefits of the introduction of technological aides [24]. The aim of this paper is to present a study protocol that will examine the efficacy of assistive technology (including home-based, mobile, and wearable technologies) in reducing executive function difficulties and care needs experienced by people with ABI.

## Methods

### Study Design

A series of SCEDs will be used to evaluate the effectiveness of the intervention technologies. The choice of design will be individualized for each participant based on the type of technology used in the intervention, the difficulty level of the behavior targeted for change, and the anticipated rate of change. It is expected that the most common design will be a withdrawal A_1_-B_1_-A_2_-B_2_ design (where A denotes the baseline phase and B denotes the intervention phase). However, multiple baseline, alternating treatments, changing criterion, and combination designs may also be used [25]. Decisions regarding the design to be implemented will be made on a case-by-case basis by the research team.

Each SCED will be planned using the Risk of Bias in N-of-1 Trials Scale [26,27] to maximize internal and external validity and will be conducted over approximately a 12-month period. The Risk of Bias in N-of-1 Trials Scale is a 15-item critical appraisal tool that evaluates the methodological quality of intervention studies using a single-case methodology. It comprises 2 subscales: internal validity and external validity and interpretation.

The report of each case will be prepared in accordance with the SCRIBE (Single-Case Reporting Guideline in Behavioral Interventions) statement [28] using a customized Microsoft Excel spreadsheet (Multimedia Appendix 1) to enable electronic record keeping. The SCRIBE is a 26-item reporting guideline developed in the CONSORT (Consolidated Standards of Reporting Trials) tradition to facilitate clear, complete, and transparent reporting of the study.

### Ethics Approval, Informed Consent, and Participation

The study design, recruitment, and data collection procedures were approved by the South Western Sydney Local Health District and Monash University Human Research Ethics Committees (Project IDs 2019/ETH14038 and 27923). The trial has been registered with the Australian New Zealand Clinical Trials Registry (ACTRN12622000835741). All participants will provide written informed consent before participating in the study. Each phase of the study will be monitored closely, and—as detailed in the procedures section—a customized Microsoft Excel spreadsheet (Multimedia Appendix 1) will be used to document and manage any adverse events or harms.

### Participant Eligibility

The inclusion criteria are as follows: participants will be aged ≥18 years, experience executive dysfunction following ABI, have substantial support needs for everyday living, reside in community dwellings, and have sufficient cognitive capacity to provide informed consent (determined by a review of any available neuropsychological assessment and/or in consultation with their treating allied health professionals). Participants will not be excluded based on their living location (metropolitan vs regional) or living situation (alone or with others). Exclusion criteria included people with current severe mental health problems or challenging behaviors.

Standardized clinical measures will be used to screen for eligibility. The instruments are described in Multimedia Appendix 2 [29-39]. The Frontal Systems Behavior Scale (FrSBe) [40] will be used to screen for executive dysfunction and the Care and Needs Scale [41] will be used for support needs. The Health of the Nation Outcome Scale—Acquired Brain Injury [42] will be used to identify severe mental health problems, and the Overt Behavior Scale–Adult [33] will be used for challenging behaviors.

### Setting

All data collection and assistive technology interventions of consenting participants will be carried out in the community, either their place of residence or work environment.

### Measures and Materials

#### Overview

All screening and outcome measures are described in Multimedia Appendix 2, along with two contextual measures: (1) Assessing Needs and Supports in Relation to Assistive Technology (A-AT; an unpublished semistructured interview) and (2) the World Health Organization Disability Assessment Schedule [34]. These will be administered to better understand participants’ functions, goals, and use of assistive technology. All published instruments have been used extensively in previous research in the field of ABI.

#### Primary Outcome Measure

##### Measures of Target Behavior(s)

In consultation with each individual participant, target behavior(s) will be identified based on their personal goal(s) for executive function support and/or participation in activities within home and community settings. The target behavior(s) will be tailored to each participant, and, as such, the measurement of the behavior will vary among participants. Target behaviors will be very specific, for example, frequency of occurrence or duration of a behavior, number of steps of an activity completed, or number of prompts from a support person. The frequency of measurement of the target behavior(s) will vary depending on the nature of the target behavior(s), but they will be measured, as appropriate, continuously throughout the baseline and intervention phases.

##### Goal Attainment Scaling

The Goal Attainment Scaling will be used to evaluate the level of goal attainment linked to the identified target behavior. Personalized evaluation scales ranging from +2 to −2 will be developed based on the framework of Krasny-Pacini et al [43] to ensure that the scales are a valid, reliable, and meaningful outcome measure.

#### Secondary Outcome Measures

The reason for including secondary measures is for the purpose of generalization and to determine whether the intervention has had effects on broader aspects of the participant’s functioning. The secondary outcome measures are as illustrated in the following sections.

##### Community Integration Questionnaire-Revised

The Community Integration Questionnaire-Revised [44] is an 18-item scale, which will be used to measure changes in community integration across four domains: (1) home integration (eg, meal preparation and housework), (2) social integration (eg, shopping, visiting friends, and leisure activities), (3) productive activity (eg, full-time vs part-time work, school, and volunteer activities), and (4) electronic social networking enabled social integration.

##### EuroQol-5 Dimensions Instrument

The self-complete (participant rated) and/or proxy (completed by a family member or paid support person(s) (ie, support worker, house manager, and allied health professional) versions of the EuroQol-5 Dimensions Instrument (EQ-5D-5L) [45] will provide a profile of health-related quality of life for the participant. The questionnaire has two components: (1) health state description and (2) evaluation. In the description section, they will be asked to rate 5 dimensions of health (mobility, self-care, usual activities, pain or discomfort, and anxiety or depression). In the evaluation part, they will be asked to indicate their overall health status (“TODAY”) using the visual analog scale from 0 to 100 with the end points labeled “the worst health you can imagine” to “the best health you can imagine,” respectively.

##### Psychosocial Impact of Assistive Devices Scale

The Psychosocial Impact of Assistive Devices Scale [46] will be used to measure the psychosocial effects the assistive technology device has had on the participant. It is a 26-item questionnaire, which will be used to measure the impact the assistive device has had on the participants’ sense of competence, adaptability, and self-esteem in daily life.

##### Quebec User Evaluation of Satisfaction With Technology

The Quebec User Evaluation of Satisfaction with Assistive Technology, version 2.0 [39], is a 12-item scale that will be used to measure the participants’ satisfaction with a technology device and its related services. This includes physical properties (eg, size, weight, ease of use, and effectiveness), and, if applicable, service delivery, maintenance, and follow-up services. The final part of the measure asks participants to choose the 3 assistive technology satisfaction items that are most important to them from a total of 12 items.

##### Disability Support Use and Cost

If agreed by the participant as part of the consent process, disability support costs will be sourced from an administration database of their funding organization to ascertain whether the assistive technology intervention impacted disability support use and cost. These data will be augmented with discussions with the participants (and/or their proxy) using a Participant Economic Evaluation Questionnaire (Multimedia Appendix 3) to ensure that all cost data, including informal supporter costs, have been accurately captured. Data will be gathered preintervention and after the second intervention. Where costs are not available for disability support through the funding organization, market rates will be applied, with the cost of informal care based on the Australian minimum wage [47]. All costs will be inflated to 2022-2023 by applying the Consumer Price Index [48].

### Blinding

Blinding in SCEDs refers to whether the participant, practitioner, and assessor are blind to the phase of the experiment. Blinding of participants will usually not be possible, given the nature of the intervention. However, the intervention is delivered by an assistive technology device, without the need for human involvement, thus eliminating human error and minimizing the risk of bias. Hence, for all intents and purposes, the “practitioner” (ie, the technology) can be considered blind to phase. Similarly, for each participant, the assessment data will be generated automatically from the assistive technology device, without the need for human involvement. Thus, the “assessor” (ie, the technology) can be considered blind to phase. In addition, data analysis will be performed by a statistician who will not be involved in discussions with the research group. The statistician will be blinded to all aspects of the intervention, including the specific phases in which data were collected.

### Intervention

#### Electronic Assistive Technology

**Figure 1:**
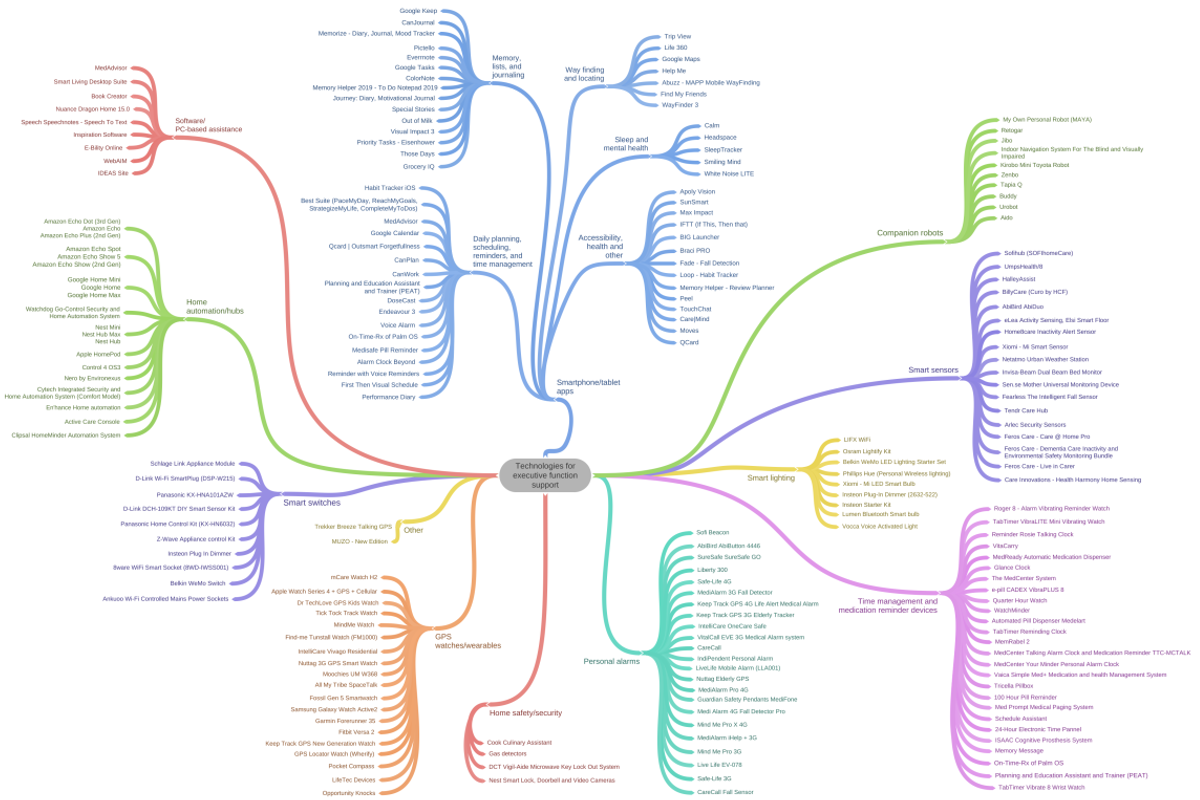
Technologies identified for use in executive function support.

A scoping review identified a wide range of technologies (eg, apps, wearables, home sensors, and smart lights) appropriate for use in executive function support [49]. These are shown in Figure 1 and will be used to shortlist electronic assistive technology appropriate for addressing the individual participant’s target behavior(s). The shortlist will be discussed with each participant, and through consultation with the participant and/or their proxy, assistive technology will be chosen for application and purchased using research funds.

#### Customized Orientation and Training Tool

Depending on the selected electronic assistive technology, there may be a need to develop a customized orientation and training tool in either a paper or digital format. The tool will include a step-by-step guide and/or video demonstration on how to navigate to prioritized function(s) that may be controlled by electronic assistive technology. Digital information will be saved to the smartphone or tablet that the participant currently uses, and written information will be left in a prominent location in the participants’ place of residence or work environment. This will ensure that they (or, where appropriate, a family member or other support person) can refer back to it if and when required.

### Procedures

#### Initial Study Setup

The initial study setup phase with the individual participants will involve several tasks, as described below. To ensure anonymity, a pseudonym will be allocated to each participant, and no identifying information (eg, residential address) will be recorded.

#### Recruitment

A third-party recruitment strategy will be used to recruit up to 10 adult participants across Victoria and New South Wales in Australia. A single-page information sheet about the study, including inclusion criteria, and a “consent to contact” form will be prepared and distributed (in hard copy or via email) to community-based allied health professionals, with the request to pass the information sheet to potentially eligible clients. The consent to contact form asks whether the individual is willing to provide their own name and phone number and the contact details of the allied health professional(s) who work with them. If the individual agrees to provide contact information, the completed form can be posted or a copy emailed to the research group. Once returned, a member of the research group will make contact to invite the individual (and/or their proxy) to a face-to-face meeting to discuss their potential participation in the research.

#### Consent to Participate

At the first meeting, a plain English information sheet of the study and consent form will be provided and explained verbally to the participant (and/or their proxy), as will the screening process, to ascertain eligibility. The individual can either choose to review the explanatory statement and consent form during that meeting and provide written consent at that time or later. If they wish to decide later, a member of the research group will email or call the individual (and/or their proxy) within 2 weeks of the meeting to see if they would like to consent to participate. This is the only follow-up contact made with the individual unless they request additional time to make their decision.

#### Screening for Eligibility

Individuals who consent to participate in the study will attend a further appointment to complete the screening assessment. Except for 1 proprietary measure (FrSBe), responses to the other measures will be recorded on a Microsoft Excel spreadsheet (Multimedia Appendix 4). Measures consist of the semistructured interview (A-AT), a clinician-rated contextual measure of functional ability (World Health Organization Disability Assessment Schedule 2.0), and 3 clinician-rated and 1 proxy-rated measures to ensure participants meet the clinical threshold (as detailed in Multimedia Appendix 2) in terms of executive function (FrSBe), have high support needs (Care and Needs Scale), and do not have mental health issues (Health of the Nation Outcome Scale—Acquired Brain Injury) or severe levels of challenging behavior (Overt Behavior Scale–Adult). Individuals who do not meet the study inclusion criteria will be thanked for their interest and time but have no further participation in the study.

### Identification of Goals, Target Behaviors, and the Intervention

#### Overview

Several sessions will be spent working with the participants (and their proxy) to identify the goal(s), target behavior(s), and intervention to be trialed in the study. As recommended, goal(s) and target behaviors will be specific, measurable, attainable, realistic, and time limited (SMART) [50,51]. Details regarding the primary outcome (target behavior) measures will be recorded on the SCRIBE checklist: Electronic Version of Selected Items (5-21; Multimedia Appendix 1). The checklist is an interactive, fillable Excel document containing 17 of the 26 items (items 5-21) detailed in the SCRIBE guidelines [28]. A total of 2 SCRIBE items were expanded from the original SCRIBE checklist by the authors of this paper: Item 14 (measures) has added Goal Attainment Scaling (which is commonly used in neurorehabilitation) to the target behaviors to measure progress toward the goal(s); item 21 (adverse events) was slightly modified to incorporate a plan or procedure to manage adverse events. A video demonstrating the use of the SCRIBE Checklist: Electronic Version of Selected Items (5-21) is available from the authors on reasonable request.

If agreed by the participant as part of the consent process, a member of the research group will take photographs of the participant’s home or work environment. The photographs will be used to help with discussions with the research team about the potential considerations regarding the layout of the house or work environment for possible technology use. A shortlist of potential assistive technologies that are appropriate to address the target behavior(s) will be developed and chosen in consultation with the participant (and/or their proxy) and the research group.

#### Phase 1: Baseline (A) Phase

Preintervention (secondary) measures will be administered (Multimedia Appendices 2 and 4). In addition, disability support costs will be sourced (Multimedia Appendix 3). During the baseline phase, the target behavior(s) will be measured repeatedly before implementing the intervention. Accordingly, this phase will serve as the control condition and include a minimum of 5 data points. For some technologies, it may be possible to use system-generated log reports to generate the target behavior data during the baseline (and intervention) phases. As such, the technology will be set up or installed (but during the baseline phase, it will not be activated, ie, for reminders or prompts) by either the occupational therapist to the research group or an external provider. If system-generated log reports are not possible, behavioral recordings of the target behavior(s) will be used.

#### Phase 2: Intervention (B) Phase

The technology will be installed, worn, and/or activated by an occupational therapist in the research group or an external provider. If required, participants (and/or their proxy) will receive a single 1-hour orientation and training session by a member of the research group on how to use the technology. Any barriers to technology use will also be identified and ameliorated if possible. The target behavior will be measured continuously throughout the intervention phase, with a minimum of 5 data points.

Depending on the specific study design for each participant, there will be subsequent phases (for A-B-A-B withdrawal designs), subphases of the intervention (for changing criterion designs), staggered introduction of the intervention across multiple target behaviors (for multiple baseline designs), or alternating interventions in rapid succession (for alternating-treatment designs). Combination designs can also be used for this purpose.

#### Postintervention Assessment

Following the completion of the intervention, the secondary outcome measures will be readministered (Multimedia Appendices 2 and 4). In addition, the disability support costs will be sourced (Multimedia Appendix 3). The occupational therapist in the research group will also discuss with the participant whether they wish to continue or cease using the technology following trial completion. If the participant chooses to cease using the technology, the occupational therapist will either uninstall the technology or organize for an external provider to do so at a mutually convenient time.

## Results

Recruitment began in June 2022 but has been impacted by the unprecedented COVID-19 pandemic. The data collection is expected to be completed by March 2024. Only data from participants who complete all baseline and intervention phases of the study will be included in the analyses; however, reasons for withdrawal will be documented (eg, hospitalization due to deteriorating health).

Participant raw data from the publicly available contextual, secondary outcome, and screening measures will be entered into a Microsoft Excel spreadsheet (Multimedia Appendix 4). The spreadsheet contains formulas and calculations that automatically calculate scores and subscores (as per author guidelines for each measure). Test booklets will be used to enter and score the participant data from the FrSBe.

The target behavior data collected during the baseline and intervention phases will be entered or imported into SPSS statistical software (version 29; IBM Corp). Data for each participant will be evaluated separately using both structured visual analyses and statistical techniques suitable for within-subject, time-series data. Structured visual analysis will use the standard protocol of Kratochwill et al [52]. It will focus on important features of the data making both within- and between-phase comparisons for response level, trend, variability, immediacy, consistency, and overlap. The Tau-U technique [53] or another suitable technique (depending on the individual data patterns) will be used to statistically quantify the overlap and generate effect sizes. The results will offer new insights into the application of various electronic assistive technologies for executive function support following ABI.

The SCED data analysis will be coupled with an exploratory economic evaluation analysis using Microsoft Excel (version 2306) and SPSS Statistics, delivered under the supervision of a health economist from the research team. This analysis will be completed by comparing a new intervention (ie, the assistive technology product applied during the intervention phase) against an alternative or comparator. In this study, the comparator is an intervention that would usually apply to the care of the population of interest in the absence of new technology, often referred to as standard care (ie, baseline phase). In addition, to ascertain whether the intervention had an impact on quality of life, changes in quality-adjusted life years (QALYs) will be compared between baseline and follow-up for both the participants and/or their informal support person.

The exploratory economic evaluation will determine both the incremental effect and incremental costs of the new technology intervention over those of the comparator state for each single case (each participant). The incremental costs will be presented as a “return on investment,” with the return referring to the change in cost for care needs and the investment referring to the cost of the assistive technology. The incremental effect will be presented as QALYs, derived from the baseline and follow-up EuroQol-5 Dimensions Instrument utility indices. The cost difference between the “return” and “investment,” together with the QALYs, will produce an individual incremental cost-effectiveness ratio for each participant.

## Discussion

### Expected Findings

This protocol for a series of single-case experiments delivered across 2 states of Australia (Victoria and New South Wales) offers digital tools, including the SCRIBE checklist: Electronic Version of Selected Items (5-21; Multimedia Appendix 1), a semistructured interview schedule (A-AT), and a customizable Microsoft Excel spreadsheet of screening tools and outcome measures (Multimedia Appendix 4). The single-case experiments will rigorously test the effectiveness of a suite of smart home, mobile, and wearable technologies and deliver high-quality evidence regarding technology interventions. Furthermore, an exploratory economic evaluation will determine the impact on formal and informal support use and quality of life.

This study extends the work of the Sofihub trial [15] and has been strategically designed to test the cause-effect functional relationships between the intervention (in this case, smart home, mobile technology, or wearable technologies) and its effect in changing the target behavior(s). Consequently, the results will address the current evidence and knowledge gaps for people with ABI who experience executive function issues, their formal and informal support networks (including health professionals), technology developers, smart home designers, and funders of disability services. Furthermore, the findings will be translated into a suite of freely available digital, written, and face-to-face educational resources to increase the capacity of key stakeholders to harness the potential of technology to build independence and reduce the cost of care for people who experience executive dysfunction following ABI. Dissemination of the study will also be undertaken by publishing results in peer-reviewed journals, presenting them at relevant conferences and in a final report provided to the 2 project sponsors.

### Limitations

This study has some potential limitations. First, the study is limited to English-speaking participants, which limits the generalizability of the findings to culturally and linguistically diverse groups. Second, people with severe mental health problems or challenging behaviors will be excluded from participating in the study, so it will not be possible to determine the potential application of various electronic assistive technologies for executive function support following ABI in those populations.

### Conclusions

The digital tools developed and made available as part of this protocol may be useful for other researchers to consider, or draw upon, when designing single-case experiments. Evaluation evidence gained from a series of SCEDs will offer new insights into the application of various electronic assistive technologies for people who experience executive dysfunction following ABI. Furthermore, the results will help increase the capacity of key stakeholders to harness the potential of technology to build independence and reduce the cost of care for this population.

## Appendix

Multimedia Appendix 1 SCRIBE (Single-Case Reporting Guideline in Behavioral Interventions) checklist: Electronic Version of Selected Items (5-21).

Multimedia Appendix 2 Summary of measures and data collection procedures.

Multimedia Appendix 3 Participant Economic Evaluation Questionnaire.

Multimedia Appendix 4 Screening tool and outcome measures.

## Abbreviations

A-AT: Assessing Needs and Supports in Relation to Assistive Technology
ABI: acquired brain injury
CONSORT: Consolidated Standards of Reporting Trials
FrSBe: Frontal Systems Behavior Scale
SCED: single-case experimental design
SCRIBE: Single-Case Reporting Guideline In BEhavioural Interventions
SMART: specific, measurable, attainable, realistic, and time limited

## Data Availability

The data sets generated during this study are not publicly available, as the study is still ongoing and analysis has not yet taken place.

## References

[1] (2007). Disability in Australia: acquired brain injury. Bulletin no. 55. Cat no. AUS 96. Australian Institute of Health and Welfare.

[2] Sloan S (2017). Understanding Acquired Brain Injury and behaviour change. ASSBI Resources.

[3] Hanks RA, Rapport LJ, Millis SR, Deshpande SA (1999). Measures of executive functioning as predictors of functional ability and social integration in a rehabilitation sample. Arch Phys Med Rehabil.

[4] Sloan S, Callaway L, Winkler D, McKinley K, Ziino C, Anson K (2012). Changes in care and support needs following community-based intervention for individuals with acquired brain injury. Brain Impairment.

[5] Spitz G, Ponsford JL, Rudzki D, Maller JJ (2012). Association between cognitive performance and functional outcome following traumatic brain injury: a longitudinal multilevel examination. Neuropsychology.

[6] Dewsbury G, Linskell J (2011). Smart home technology for safety and functional independence: the UK experience. NeuroRehabilitation.

[7] Gentry T (2009). Smart homes for people with neurological disability: state of the art. NeuroRehabilitation.

[8] Wild MR (2013). Assistive technology for cognition following brain injury: guidelines for device and app selection. Perspect Neurophysiol Neurogenic Speech Lang Disord.

[9] Wong D, Sinclair K, Seabrook E, McKay A, Ponsford J (2017). Smartphones as assistive technology following traumatic brain injury: a preliminary study of what helps and what hinders. Disabil Rehabil.

[10] De Joode EA, Van Heugten CM, Verhey FR, Van Boxtel MP (2013). Effectiveness of an electronic cognitive aid in patients with acquired brain injury: a multicentre randomised parallel-group study. Neuropsychol Rehabil.

[11] McDonald A, Haslam C, Yates P, Gurr B, Leeder G, Sayers A (2011). Google Calendar: a new memory aid to compensate for prospective memory deficits following acquired brain injury. Neuropsychol Rehabil.

[12] Culley C, Evans JJ (2010). SMS text messaging as a means of increasing recall of therapy goals in brain injury rehabilitation: a single-blind within-subjects trial. Neuropsychol Rehabil.

[13] Hart T, Hawkey K, Whyte J (2002). Use of a portable voice organizer to remember therapy goals in traumatic brain injury rehabilitation: a within-subjects trial. J Head Trauma Rehabil.

[14] Gracey F, Fish JE, Greenfield E, Bateman A, Malley D, Hardy G, Ingham J, Evans JJ, Manly T (2017). A randomized controlled trial of assisted intention monitoring for the rehabilitation of executive impairments following acquired brain injury. Neurorehabil Neural Repair.

[15] Fernando N, Vouliotis A, Calloway L (2018). Pilot evaluation of the effectiveness of a new activity sensing technology providing cognitive support in housing for people with acquired brain injury. School of Information Technology, Deakin University and Occupational Therapy Department, Monash University.

[16] Assistive technology strategy. National Disability Insurance Agency.

[17] Callaway L, Winkler D, Sloan S, Pattuwage L, Osborn W, Pitt V (2013). Models of supported accommodation for people with traumatic brain injury: a systematic review. Transport Accident Commission, Institute of Safety, Compensation and Recovery Research, and the Occupational Therapy Department at Monash University.

[18] Charters E, Gillett L, Simpson GK (2015). Efficacy of electronic portable assistive devices for people with acquired brain injury: a systematic review. Neuropsychol Rehabil.

[19] Byiers BJ, Reichle J, Symons FJ (2012). Single-subject experimental design for evidence-based practice. Am J Speech Lang Pathol.

[20] Krasny-Pacini A, Evans J (2018). Single-case experimental designs to assess intervention effectiveness in rehabilitation: a practical guide. Ann Phys Rehabil Med.

[21] Lane JD, Ledford JR, Gast DL (2017). Single-case experimental design: current standards and applications in occupational therapy. Am J Occup Ther.

[22] Perdices M, Tate RL, Rosenkoetter U (2019). An algorithm to evaluate methodological rigor and risk of bias in single-case studies. Behav Modif.

[23] Tate RL, Perdices M, McDonald S, Togher L, Rosenkoetter U (2014). The design, conduct and report of single-case research: resources to improve the quality of the neurorehabilitation literature. Neuropsychol Rehabil.

[24] Bridge C, Zmudzki F, Huang T, Owen C, Faulkner D (2021). Impacts of new and emerging assistive technologies for ageing and disabled housing. Australian Housing and Urban Research Institute Limited.

[25] Tate R, Perdices M (2018). Single-Case Experimental Designs for Clinical Research and Neurorehabilitation Settings Planning, Conduct, Analysis and Reporting.

[26] Tate RL, Perdices M, Rosenkoetter U, Wakim D, Godbee K, Togher L, McDonald S (2013). Revision of a method quality rating scale for single-case experimental designs and n-of-1 trials: the 15-item Risk of Bias in N-of-1 Trials (RoBiNT) Scale. Neuropsychol Rehabil.

[27] Tate RL, Rosenkoetter U, Wakim D, Sigmundsdottir L, Doubleday J, Togher L, Mcdonald S, Perdices M (2015). The Risk of Bias in N-of-1 Trials (RoBiNT) Scale: an expanded manual for the critical appraisal of single-case reports. PsycBITE Group.

[28] Tate RL, Perdices M, Rosenkoetter U, McDonald S, Togher L, Shadish W, Horner R, Kratochwill T, Barlow DH, Kazdin A, Sampson M, Shamseer L, Vohra S (2016). The Single-Case Reporting Guideline In BEhavioural Interventions (SCRIBE) 2016: explanation and elaboration. Arch Sci Psychol.

[29] Niemeier JP, Perrin PB, Holcomb MG, Nersessova KS, Rolston CD (2013). Factor structure, reliability, and validity of the Frontal Systems Behavior Scale (FrSBe) in an acute traumatic brain injury population. Rehabil Psychol.

[30] Soo C, Tate R, Hopman K, Forman M, Secheny T, Aird V, Browne S, Coulston C (2007). Reliability of the care and needs scale for assessing support needs after traumatic brain injury. J Head Trauma Rehabil.

[31] Wing JK, Beevor AS, Curtis RH, Park SB, Hadden S, Burns A (1998). Health of the Nation Outcome Scales (HoNOS). Research and development. Br J Psychiatry.

[32] Sabaz M, Simpson GK, Walker AJ, Rogers JM, Gillis I, Strettles B (2014). Prevalence, comorbidities, and correlates of challenging behavior among community-dwelling adults with severe traumatic brain injury: a multicenter study. J Head Trauma Rehabil.

[33] Kelly G, Todd J, Simpson G, Kremer P, Martin C (2006). The Overt Behaviour Scale (OBS): a tool for measuring challenging behaviours following ABI in community settings. Brain Inj.

[34] Üstün TB, Kostanjsek N, Chatterji, S, Rehm J (2010). Measuring health and disability: manual for WHO Disability Assessment Schedule WHODAS 2.0. World Health Organization.

[35] Cytrynbaum S, Ginath Y, Birdwell J, Brandt L (2016). Goal attainment scaling: a critical review. Eval Q.

[36] Callaway L, Winkler D, Tippett A, Migliorini C, Herd N, Willer B (2014). The Community Integration Questionnaire-Revised (CIQ-R). Summer Foundation Ltd.

[37] Long D, Polinder S, Bonsel GJ, Haagsma JA (2021). Test-retest reliability of the EQ-5D-5L and the reworded QOLIBRI-OS in the general population of Italy, the Netherlands, and the United Kingdom. Qual Life Res.

[38] Atigossou OL, Honado AS, Routhier F, Flamand VH (2023). Psychometric properties of the psychosocial impact of assistive devices scale (PIADS): a systematic review. Assist Technol.

[39] Demers L, Weiss-Lambrou R, Ska B (2002). The Quebec User Evaluation of Satisfaction with Assistive Technology (QUEST 2.0): an overview and recent progress. Technol Disabil.

[40] Grace J, Malloy P Frontal systems behavior scale™. PAR Inc.

[41] Tate RL (2017). Manual for the Care and Needs Scale (CANS). John Walsh Centre for Rehabilitation Research University of Sydney.

[42] Fleminger S, Leigh E, Eames P, Langrell L, Nagraj R, Logsdail S (2018). HoNOS–ABI: a reliable outcome measure of neuropsychiatric sequelae to brain injury?. Psychiatr Bull.

[43] Krasny-Pacini A, Evans J, Sohlberg MM, Chevignard M (2016). Proposed criteria for appraising goal attainment scales used as outcome measures in rehabilitation research. Arch Phys Med Rehabil.

[44] Callaway L, Winkler D, Tippett A, Herd N, Migliorini C, Willer B (2016). The Community Integration Questionnaire - Revised: Australian normative data and measurement of electronic social networking. Aust Occup Ther J.

[45] Devlin NJ, Brooks R (2017). EQ-5D and the EuroQol group: past, present and future. Appl Health Econ Health Policy.

[46] Jutai J, Day H (2002). Psychosocial impact of assistive devices scale (PIADS). Technol Disabil.

[47] Minimum wages. Fair Work Ombudsman. Australian Government.

[48] Consumer price index, Australia. Australian Bureau of Statistics.

[49] Liddle J, Callaway L, Simpson G Electronic assistive technology to support executive functioning: A scoping review focusing on clinical implications.

[50] Evans JJ, Krasny-Pacini A, Wilson BA, Winegardner J, van Heugten CM, Ownsworth T (2017). Goal setting in rehabilitation. Neuropsychological Rehabilitation: The International Handbook.

[51] Turner-Stokes L (2009). Goal attainment scaling (GAS) in rehabilitation: a practical guide. Clin Rehabil.

[52] Kratochwill TR, Hitchcock JH, Horner RH, Levin JR, Odom SL, Rindskopf DM, Shadish WR (2012). Single-case intervention research design standards. Remedial Special Educ.

[53] Parker RI, Vannest KJ, Davis JL, Sauber SB (2011). Combining nonoverlap and trend for single-case research: Tau-U. Behav Ther.

