# Supplementary material for: Evaluation of the Effectiveness of Assistive Technology for Executive Function Support for People With Acquired Brain Injury: Protocol for Single-Case Experimental Designs
Source: JMIR Res Protoc. 2023 Aug 29;12:e48503. doi: 10.2196/48503 (PMC10498320; doi:10.2196/48503)
Supplement: Multimedia Appendix 2 [file resprot_v12i1e48503_app2.pdf]

Summary of measures and data collection procedures.

	Measure	Response format	Scoring procedure, score, and range	Score interpretation and exclusion criteria (where applicable /available)	Selected psychometric properties	Rater(s)	Time point(s)	Repeat measure
**Screening and contextual**	Frontal Systems Behavior Scale (FrSBe)	1= Almost never to 5= Almost always	Test booklet used to calculate score across three subscales (Apathy, Disinhibition and Executive Dysfunction).	Scores converted into standardized T-scores (M=50, SD=10) to provide a FrSBe caseness score.<br><br>Exclusion criteria: T-score of 64 or less on all three subscales.	Test–retest reliability – family (average time 7.24 days): r =.72 [29]	Proxy	Baseline screening	Once
	Care and Needs Scale (CANS)	Section 1: 28 items are endorsed if a support need is present.<br><br>Section 2 Support level: 0 = does not need contact to 7 = cannot be left alone.	Excel spreadsheet used.<br><br>Section 1: scores are summed (range 0-28).<br>Section 2: classification into one of eight levels.	Higher scores indicate greater support needs.<br><br>Exclusion criteria: Support level lower than 4 on section 2.	Test–retest reliability (1 week): ICC[a] = .98 [30].	Clinician-rated with participant	Baseline screening	Once

Measure	Response format	Scoring procedure, score, and range	Score interpretation and exclusion criteria (where applicable /available)	Selected psychometric properties	Rater(s)	Time point(s)	Repeat measure
Health of the Nation Outcome Scale – Acquired Brain Injury (HoNOS-ABI)	0= No problem to 4= Severe problem.	Excel spreadsheet used.<br><br>12-items are summed to obtain a total score (range 0-48)	Higher scores indicate poorer functioning.<br><br>Exclusion criteria: Severe problem on either items: self-directed injury; problem drinking or drug use; problems with hallucinations/ delusions/ confabulation; problems with depressive symptoms; other mental and behavioral problems.	Test–retest reliability (35 days): ICC = .82 (range .61-.88 [31]	Clinician-rated with participant	Baseline screening	Once
Overt Behavior Scale–Adult (OBS-A)	Frequency 0= nil to 5= multiple per daily<br><br>Impact 1 = no impact to 5 = extreme impact	Excel spreadsheet used.<br><br>Sum frequency of behavior for the 9 subscales to obtain a total	Higher scores indicate poorer functioning.<br><br>Exclusion criteria: Meet the classification for ‘challenging’ [32].	Test–retest reliability (1 week): r = .72–.77 [33].	Clinician-rated with participant	Baseline screening	Once

	Measure	Response format	Scoring procedure, score, and range	Score interpretation and exclusion criteria (where applicable /available)	Selected psychometric properties	Rater(s)	Time point(s)	Repeat measure
			score (range 0-84)					
	Semi-structured interview (A-AT)	Open and closed ended questions to collect: (1) demographic data; (2) type(s) of natural, paid, and cognitive aides they currently use for executive function support; (3) what they look for / avoid in relation to assistive technology; and (4) the kind of support they would like when trialing new assistive technology.	Excel spreadsheet used.	Data used to ascertain the participants' goal(s) for executive function support / participation in activities within home and community settings.	Not established.	Clinician-rated with participant	Baseline screening	Once

	Measure	Response format	Scoring procedure, score, and range	Score interpretation and exclusion criteria (where applicable /available)	Selected psychometric properties	Rater(s)	Time point(s)	Repeat measure
	World Health Organization Disability Assessment Schedule (WHODAS 2.0)	1=None to 5= Extreme or cannot do	Excel spreadsheet used.<br><br>36-items are scored (using the item-response theory) across six life domains to give a global disability score (range 0 to 100).	0= no disability to 100= full disability.	Test–retest reliability (mean interval 2.4 ± 1.6 days): ICC = .69 – .89 [34]	Clinician-rated with participant	Baseline screening	Once
**Primary outcome**	Measurement of Target Behavior(s)	Target behavior(s) – tailored to each participant	Tailored to each participant.	Comparison of baseline and intervention data	Not applicable.	Proxy Or system-generated log reports	Throughout all phases	Multiple times
	Goal Attainment Scaling (GAS)	-2 to +2	Each goal will be measured to represent level of attainment.	0= expected outcome was achieved, -1 to -2 expected outcome was achieved much less, +1 to +2 greater than expected outcome was achieved.	Test–retest reliability (3 weeks and 2 months) r =.70 [35]	Clinician-rated with participant (and/or proxy)	Throughout all phases	Multiple times

	Measure	Response format	Scoring procedure, score, and range	Score interpretation and exclusion criteria (where applicable /available)	Selected psychometric properties	Rater(s)	Time point(s)	Repeat measure
**Secondary outcome**	Community Integration Questionnaire -Revised (CIQ-R)	Frequency for each item provides a score ranging from 0-2.	Excel spreadsheet used.<br><br>Sum scores from 18-items to obtain sub-scores across the four domains (range 6-12) and total score (range 0-25).	Higher scores indicate greater independence and community integration.	Test–retest reliability (10 weeks) r =.84 [36]	Clinician-rated with participant	Pre- and post-intervention	Twice
	EuroQol-5 Dimensions Instrument (EQ-5D-5L)	EQ-5D-5L utility: 0= no problems to 5= extreme problems.<br><br>EQ-VAS: 0 (worst health) to 100 (best health).	Excel spreadsheet used.<br><br>Health state description: raw scores converted into a utility index (range 0-1)	Negative score = a state worse than death; 0= state of health equal to death, and 1= perfect state of health.	Test–retest reliability (7-8 months): EQ-5D-5L summary index ICC =.73 – .84; EQVAS ICC =.61 – .68 [37]	Participant-rated and/or proxy-rated	Pre- and post-intervention	Twice

Measure	Response format	Scoring procedure, score, and range	Score interpretation and exclusion criteria (where applicable /available)	Selected psychometric properties	Rater(s)	Time point(s)	Repeat measure
Psychosocial Impact of Assistive Devices Scale (PIADS)	26-items are rated on how the device affects the user from -3 (negatively impacts the user) to +3 (positively impacts the user). 0= no perceived impact to the user.	Excel spreadsheet used.<br><br>Three subscale scores are calculated for competence, adaptability, and self-esteem.	Higher score indicates greater psychosocial effects of the assistive technology.	Test–retest reliability (1-2 weeks): ICC =.76 – .96 [38]	Participant-rated and/or proxy-rated	Post-intervention	Once
Quebec User Evaluation of Satisfaction with Assistive Technology (QUEST)	Rate device: 1= not satisfied at all to 5= very satisfied.<br><br>Items most important: Choose three from a list of 12 items.	Excel spreadsheet used.<br><br>Responses summed and divided by number of valid responses to obtain a total score and two sub-scores (range 1-5).	Higher score indicates greater satisfaction with assistive technology.	Test–retest reliability (7-10 days) ICC =.82 (Device); .82 (Services); .91 (Total) [39]	Participant-rated and/or proxy-rated	Post-intervention	Once

Measure	Response format	Scoring procedure, score, and range	Score interpretation and exclusion criteria (where applicable /available)	Selected psychometric properties	Rater(s)	Time point(s)	Repeat measure
Disability support utilization and cost	Disability support costs obtained for a maximum of three months.	Funding organization administration database and Customer Economic Evaluation Questionnaire (see Multimedia Appendix 4).<br>Costs summed to obtain total disability support costs.	Change over time will ascertain whether the assistive technology intervention impacted disability support utilization and cost.	Not established	Funding organization administration database and clinician-rated with participant (and/or proxy)	Pre- and post-intervention	Twice

[a]ICC: Intraclass coefficient.
