# Supplementary material for: Evaluation of the Effectiveness of Assistive Technology for Executive Function Support for People With Acquired Brain Injury: Protocol for Single-Case Experimental Designs
Source: JMIR Res Protoc. 2023 Aug 29;12:e48503. doi: 10.2196/48503 (PMC10498320; doi:10.2196/48503)
Supplement: Multimedia Appendix 3 [file resprot_v12i1e48503_app3.docx]

**Participant Economic Evaluation Questionnaire**

1. Ask the participant (and/or their proxy) to reflect on the last two weeks, and think about all the support / service (both paid/formal disability supports and unpaid/informal support provided by family etc) they have received that is related to / required because of their acquired brain injury (ABI).
2. Walk through the table below with the participant (and/or their proxy) by completing the following steps:
   1. Complete the table for each day of the week
   2. Once you complete week 1, ask if it is reflective of week 2
      1. if yes, only complete week 1 then tick this box
      2. if no, repeat for week 2
3. Ask if the last 2 weeks reflect (on average) the last 3 months? Does the person with ABI usually have more or less support / services than what has been described here? Please provide details.
   1. Details:
4. During the 2^nd^ questionnaire (following the intervention), ask if there has been any change to the household income since the 1^st^ questionnaire. If so, please ask who had the change (e.g. participant, parent, spouse), and for an indication of the weekly change in income, (some people may prefer to just state it is higher or lower following the intervention).
   1. Details for the participant:
      1. No change in income then tick this box
      2. Change in income (details, e.g. more/less hours, wages):
   2. Details for other people living in the house:
      1. No change in income then tick this box
      2. Change in income (details, e.g. who, more/less hours, wages):

| **Monday – Week 1** | | | | | | |
| --- | --- | --- | --- | --- | --- | --- |
| **Goal Activity:** | | | | | | |
| 1. On a Monday, do you receive support? | Yes  No (move on to the next day) | | | | | |
| 1. What support? | Does this support relate to the **goal activity**? | How many hours was the support / service? | Paid travel time for the support person (only include if this is paid time, e.g. travel time for a physio to come to your house)? | Your travel time for the support / service? | Did a carer attend with you? If so, what was the carer time (e.g. driving the participant to an appointment – include the travel time) | How was this support / service paid? |
| Unpaid disability support / carer (e.g. family / friends; helping with the morning routine) | Yes  No |  |  |  |  | N/A |
| Paid disability support / carer | Yes  No |  |  |  |  | Privately  Disability / injury insurer  Public health  Other |
| Allied health / mental health / employment support | Yes  No |  |  |  |  | Privately  Disability / injury insurer  Public health  Other |
| Recreational & social support / programs | Yes  No |  |  |  |  | Privately  Disability / injury insurer  Public health  Other |
| Medical care or nursing, pathology, imaging | Yes  No |  |  |  |  | Privately  Disability / injury insurer  Public health  Other |
| Other (e.g., pensions, allowances, assistive technology) Details: | Yes  No |  |  |  |  | Privately  Disability / injury insurer  Public health  Other |

| **Tuesday – Week 1** | | | | | | |
| --- | --- | --- | --- | --- | --- | --- |
| **Goal Activity:** | | | | | | |
| 1. On a Tuesday, do you receive support? | Yes  No (move on to the next day) | | | | | |
| 1. What support? | Does this support relate to the **goal activity**? | How many hours was the support / service? | Paid travel time for the support person (only include if this is paid time, e.g. travel time for a physio to come to your house)? | Your travel time for the support / service? | Did a carer attend with you? If so, what was the carer time (e.g. driving the participant to an appointment – include the travel time) | How was this support / service paid? |
| Unpaid disability support / carer (e.g. family / friends; helping with the morning routine) | Yes  No |  |  |  |  | N/A |
| Paid disability support / carer | Yes  No |  |  |  |  | Privately  Disability / injury insurer  Public health  Other |
| Allied health / mental health / employment support | Yes  No |  |  |  |  | Privately  Disability / injury insurer  Public health  Other |
| Recreational & social support / programs | Yes  No |  |  |  |  | Privately  Disability / injury insurer  Public health  Other |
| Medical care or nursing, pathology, imaging | Yes  No |  |  |  |  | Privately  Disability / injury insurer  Public health  Other |
| Other (e.g., pensions, allowances, assistive technology) Details: | Yes  No |  |  |  |  | Privately  Disability / injury insurer  Public health  Other |

| **Wednesday – Week 1** | | | | | | |
| --- | --- | --- | --- | --- | --- | --- |
| **Goal Activity:** | | | | | | |
| 1. On a Wednesday, do you receive support? | Yes  No (move on to the next day) | | | | | |
| 1. What support? | Does this support relate to the **goal activity**? | How many hours was the support / service? | Paid travel time for the support person (only include if this is paid time, e.g. travel time for a physio to come to your house)? | Your travel time for the support / service? | Did a carer attend with you? If so, what was the carer time (e.g. driving the participant to an appointment – include the travel time) | How was this support / service paid? |
| Unpaid disability support / carer (e.g. family / friends; helping with the morning routine) | Yes  No |  |  |  |  | N/A |
| Paid disability support / carer | Yes  No |  |  |  |  | Privately  Disability / injury insurer  Public health  Other |
| Allied health / mental health / employment support | Yes  No |  |  |  |  | Privately  Disability / injury insurer  Public health  Other |
| Recreational & social support / programs | Yes  No |  |  |  |  | Privately  Disability / injury insurer  Public health  Other |
| Medical care or nursing, pathology, imaging | Yes  No |  |  |  |  | Privately  Disability / injury insurer  Public health  Other |
| Other (e.g., pensions, allowances, assistive technology) Details: | Yes  No |  |  |  |  | Privately  Disability / injury insurer  Public health  Other |

| **Thursday – Week 1** | | | | | | |
| --- | --- | --- | --- | --- | --- | --- |
| **Goal Activity:** | | | | | | |
| 1. On a Thursday, do you receive support? | Yes  No (move on to the next day) | | | | | |
| 1. What support? | Does this support relate to the **goal activity**? | How many hours was the support / service? | Paid travel time for the support person (only include if this is paid time, e.g. travel time for a physio to come to your house)? | Your travel time for the support / service? | Did a carer attend with you? If so, what was the carer time (e.g. driving the participant to an appointment – include the travel time) | How was this support / service paid? |
| Unpaid disability support / carer (e.g. family / friends; helping with the morning routine) | Yes  No |  |  |  |  | N/A |
| Paid disability support / carer | Yes  No |  |  |  |  | Privately  Disability / injury insurer  Public health  Other |
| Allied health / mental health / employment support | Yes  No |  |  |  |  | Privately  Disability / injury insurer  Public health  Other |
| Recreational & social support / programs | Yes  No |  |  |  |  | Privately  Disability / injury insurer  Public health  Other |
| Medical care or nursing, pathology, imaging | Yes  No |  |  |  |  | Privately  Disability / injury insurer  Public health  Other |
| Other (e.g., pensions, allowances, assistive technology) Details: | Yes  No |  |  |  |  | Privately  Disability / injury insurer  Public health  Other |

| **Friday – Week 1** | | | | | | |
| --- | --- | --- | --- | --- | --- | --- |
| **Goal Activity:** | | | | | | |
| 1. On a Friday, do you receive support? | Yes  No (move on to the next day) | | | | | |
| 1. What support? | Does this support relate to the **goal activity**? | How many hours was the support / service? | Paid travel time for the support person (only include if this is paid time, e.g. travel time for a physio to come to your house)? | Your travel time for the support / service? | Did a carer attend with you? If so, what was the carer time (e.g. driving the participant to an appointment – include the travel time) | How was this support / service paid? |
| Unpaid disability support / carer (e.g. family / friends; helping with the morning routine) | Yes  No |  |  |  |  | N/A |
| Paid disability support / carer | Yes  No |  |  |  |  | Privately  Disability / injury insurer  Public health  Other |
| Allied health / mental health / employment support | Yes  No |  |  |  |  | Privately  Disability / injury insurer  Public health  Other |
| Recreational & social support / programs | Yes  No |  |  |  |  | Privately  Disability / injury insurer  Public health  Other |
| Medical care or nursing, pathology, imaging | Yes  No |  |  |  |  | Privately  Disability / injury insurer  Public health  Other |
| Other (e.g., pensions, allowances, assistive technology) Details: | Yes  No |  |  |  |  | Privately  Disability / injury insurer  Public health  Other |

| **Saturday – Week 1** | | | | | | |
| --- | --- | --- | --- | --- | --- | --- |
| **Goal Activity:** | | | | | | |
| 1. On a Saturday, do you receive support? | Yes  No (move on to the next day) | | | | | |
| 1. What support? | Does this support relate to the **goal activity**? | How many hours was the support / service? | Paid travel time for the support person (only include if this is paid time, e.g. travel time for a physio to come to your house)? | Your travel time for the support / service? | Did a carer attend with you? If so, what was the carer time (e.g. driving the participant to an appointment – include the travel time) | How was this support / service paid? |
| Unpaid disability support / carer (e.g. family / friends; helping with the morning routine) | Yes  No |  |  |  |  | N/A |
| Paid disability support / carer | Yes  No |  |  |  |  | Privately  Disability / injury insurer  Public health  Other |
| Allied health / mental health / employment support | Yes  No |  |  |  |  | Privately  Disability / injury insurer  Public health  Other |
| Recreational & social support / programs | Yes  No |  |  |  |  | Privately  Disability / injury insurer  Public health  Other |
| Medical care or nursing, pathology, imaging | Yes  No |  |  |  |  | Privately  Disability / injury insurer  Public health  Other |
| Other (e.g., pensions, allowances, assistive technology) Details: | Yes  No |  |  |  |  | Privately  Disability / injury insurer  Public health  Other |

| **Sunday – Week 1** | | | | | | |
| --- | --- | --- | --- | --- | --- | --- |
| **Goal Activity:** | | | | | | |
| 1. On a Sunday, do you receive support? | Yes  No (move on to the next day) | | | | | |
| 1. What support? | Does this support relate to the **goal activity**? | How many hours was the support / service? | Paid travel time for the support person (only include if this is paid time, e.g. travel time for a physio to come to your house)? | Your travel time for the support / service? | Did a carer attend with you? If so, what was the carer time (e.g. driving the participant to an appointment – include the travel time) | How was this support / service paid? |
| Unpaid disability support / carer (e.g. family / friends; helping with the morning routine) | Yes  No |  |  |  |  | N/A |
| Paid disability support / carer | Yes  No |  |  |  |  | Privately  Disability / injury insurer  Public health  Other |
| Allied health / mental health / employment support | Yes  No |  |  |  |  | Privately  Disability / injury insurer  Public health  Other |
| Recreational & social support / programs | Yes  No |  |  |  |  | Privately  Disability / injury insurer  Public health  Other |
| Medical care or nursing, pathology, imaging | Yes  No |  |  |  |  | Privately  Disability / injury insurer  Public health  Other |
| Other (e.g., pensions, allowances, assistive technology) Details: | Yes  No |  |  |  |  | Privately  Disability / injury insurer  Public health  Other |

| **Monday – Week 2** | | | | | | |
| --- | --- | --- | --- | --- | --- | --- |
| **Goal Activity:** | | | | | | |
| 1. On a Monday, do you receive support? | Yes  No (move on to the next day) | | | | | |
| 1. What support? | Does this support relate to the **goal activity**? | How many hours was the support / service? | Paid travel time for the support person (only include if this is paid time, e.g. travel time for a physio to come to your house)? | Your travel time for the support / service? | Did a carer attend with you? If so, what was the carer time (e.g. driving the participant to an appointment – include the travel time) | How was this support / service paid? |
| Unpaid disability support / carer (e.g. family / friends; helping with the morning routine) | Yes  No |  |  |  |  | N/A |
| Paid disability support / carer | Yes  No |  |  |  |  | Privately  Disability / injury insurer  Public health  Other |
| Allied health / mental health / employment support | Yes  No |  |  |  |  | Privately  Disability / injury insurer  Public health  Other |
| Recreational & social support / programs | Yes  No |  |  |  |  | Privately  Disability / injury insurer  Public health  Other |
| Medical care or nursing, pathology, imaging | Yes  No |  |  |  |  | Privately  Disability / injury insurer  Public health  Other |
| Other (e.g., pensions, allowances, assistive technology) Details: | Yes  No |  |  |  |  | Privately  Disability / injury insurer  Public health  Other |

| **Tuesday – Week 2** | | | | | | |
| --- | --- | --- | --- | --- | --- | --- |
| **Goal Activity:** | | | | | | |
| 1. On a Tuesday, do you receive support? | Yes  No (move on to the next day) | | | | | |
| 1. What support? | Does this support relate to the **goal activity**? | How many hours was the support / service? | Paid travel time for the support person (only include if this is paid time, e.g. travel time for a physio to come to your house)? | Your travel time for the support / service? | Did a carer attend with you? If so, what was the carer time (e.g. driving the participant to an appointment – include the travel time) | How was this support / service paid? |
| Unpaid disability support / carer (e.g. family / friends; helping with the morning routine) | Yes  No |  |  |  |  | N/A |
| Paid disability support / carer | Yes  No |  |  |  |  | Privately  Disability / injury insurer  Public health  Other |
| Allied health / mental health / employment support | Yes  No |  |  |  |  | Privately  Disability / injury insurer  Public health  Other |
| Recreational & social support / programs | Yes  No |  |  |  |  | Privately  Disability / injury insurer  Public health  Other |
| Medical care or nursing, pathology, imaging | Yes  No |  |  |  |  | Privately  Disability / injury insurer  Public health  Other |
| Other (e.g., pensions, allowances, assistive technology) Details: | Yes  No |  |  |  |  | Privately  Disability / injury insurer  Public health  Other |

| **Wednesday – Week 2** | | | | | | |
| --- | --- | --- | --- | --- | --- | --- |
| **Goal Activity:** | | | | | | |
| 1. On a Wednesday, do you receive support? | Yes  No (move on to the next day) | | | | | |
| 1. What support? | Does this support relate to the **goal activity**? | How many hours was the support / service? | Paid travel time for the support person (only include if this is paid time, e.g. travel time for a physio to come to your house)? | Your travel time for the support / service? | Did a carer attend with you? If so, what was the carer time (e.g. driving the participant to an appointment – include the travel time) | How was this support / service paid? |
| Unpaid disability support / carer (e.g. family / friends; helping with the morning routine) | Yes  No |  |  |  |  | N/A |
| Paid disability support / carer | Yes  No |  |  |  |  | Privately  Disability / injury insurer  Public health  Other |
| Allied health / mental health / employment support | Yes  No |  |  |  |  | Privately  Disability / injury insurer  Public health  Other |
| Recreational & social support / programs | Yes  No |  |  |  |  | Privately  Disability / injury insurer  Public health  Other |
| Medical care or nursing, pathology, imaging | Yes  No |  |  |  |  | Privately  Disability / injury insurer  Public health  Other |
| Other (e.g., pensions, allowances, assistive technology) Details: | Yes  No |  |  |  |  | Privately  Disability / injury insurer  Public health  Other |

| **Thursday – Week 2** | | | | | | |
| --- | --- | --- | --- | --- | --- | --- |
| **Goal Activity:** | | | | | | |
| 1. On a Thursday, do you receive support? | Yes  No (move on to the next day) | | | | | |
| 1. What support? | Does this support relate to the **goal activity**? | How many hours was the support / service? | Paid travel time for the support person (only include if this is paid time, e.g. travel time for a physio to come to your house)? | Your travel time for the support / service? | Did a carer attend with you? If so, what was the carer time (e.g. driving the participant to an appointment – include the travel time) | How was this support / service paid? |
| Unpaid disability support / carer (e.g. family / friends; helping with the morning routine) | Yes  No |  |  |  |  | N/A |
| Paid disability support / carer | Yes  No |  |  |  |  | Privately  Disability / injury insurer  Public health  Other |
| Allied health / mental health / employment support | Yes  No |  |  |  |  | Privately  Disability / injury insurer  Public health  Other |
| Recreational & social support / programs | Yes  No |  |  |  |  | Privately  Disability / injury insurer  Public health  Other |
| Medical care or nursing, pathology, imaging | Yes  No |  |  |  |  | Privately  Disability / injury insurer  Public health  Other |
| Other (e.g., pensions, allowances, assistive technology) Details: | Yes  No |  |  |  |  | Privately  Disability / injury insurer  Public health  Other |

| **Friday – Week 2** | | | | | | |
| --- | --- | --- | --- | --- | --- | --- |
| **Goal Activity:** | | | | | | |
| 1. On a Friday, do you receive support? | Yes  No (move on to the next day) | | | | | |
| 1. What support? | Does this support relate to the **goal activity**? | How many hours was the support / service? | Paid travel time for the support person (only include if this is paid time, e.g. travel time for a physio to come to your house)? | Your travel time for the support / service? | Did a carer attend with you? If so, what was the carer time (e.g. driving the participant to an appointment – include the travel time) | How was this support / service paid? |
| Unpaid disability support / carer (e.g. family / friends; helping with the morning routine) | Yes  No |  |  |  |  | N/A |
| Paid disability support / carer | Yes  No |  |  |  |  | Privately  Disability / injury insurer  Public health  Other |
| Allied health / mental health / employment support | Yes  No |  |  |  |  | Privately  Disability / injury insurer  Public health  Other |
| Recreational & social support / programs | Yes  No |  |  |  |  | Privately  Disability / injury insurer  Public health  Other |
| Medical care or nursing, pathology, imaging | Yes  No |  |  |  |  | Privately  Disability / injury insurer  Public health  Other |
| Other (e.g., pensions, allowances, assistive technology) Details: | Yes  No |  |  |  |  | Privately  Disability / injury insurer  Public health  Other |

| **Saturday – Week 2** | | | | | | |
| --- | --- | --- | --- | --- | --- | --- |
| **Goal Activity:** | | | | | | |
| 1. On a Saturday, do you receive support? | Yes  No (move on to the next day) | | | | | |
| 1. What support? | Does this support relate to the **goal activity**? | How many hours was the support / service? | Paid travel time for the support person (only include if this is paid time, e.g. travel time for a physio to come to your house)? | Your travel time for the support / service? | Did a carer attend with you? If so, what was the carer time (e.g. driving the participant to an appointment – include the travel time) | How was this support / service paid? |
| Unpaid disability support / carer (e.g. family / friends; helping with the morning routine) | Yes  No |  |  |  |  | N/A |
| Paid disability support / carer | Yes  No |  |  |  |  | Privately  Disability / injury insurer  Public health  Other |
| Allied health / mental health / employment support | Yes  No |  |  |  |  | Privately  Disability / injury insurer  Public health  Other |
| Recreational & social support / programs | Yes  No |  |  |  |  | Privately  Disability / injury insurer  Public health  Other |
| Medical care or nursing, pathology, imaging | Yes  No |  |  |  |  | Privately  Disability / injury insurer  Public health  Other |
| Other (e.g., pensions, allowances, assistive technology) Details: | Yes  No |  |  |  |  | Privately  Disability / injury insurer  Public health  Other |

| **Sunday – Week 2** | | | | | | |
| --- | --- | --- | --- | --- | --- | --- |
| **Goal Activity:** | | | | | | |
| 1. On a Sunday, do you receive support? | Yes  No (move on to the next day) | | | | | |
| 1. What support? | Does this support relate to the **goal activity**? | How many hours was the support / service? | Paid travel time for the support person (only include if this is paid time, e.g. travel time for a physio to come to your house)? | Your travel time for the support / service? | Did a carer attend with you? If so, what was the carer time (e.g. driving the participant to an appointment – include the travel time) | How was this support / service paid? |
| Unpaid disability support / carer (e.g. family / friends; helping with the morning routine) | Yes  No |  |  |  |  | N/A |
| Paid disability support / carer | Yes  No |  |  |  |  | Privately  Disability / injury insurer  Public health  Other |
| Allied health / mental health / employment support | Yes  No |  |  |  |  | Privately  Disability / injury insurer  Public health  Other |
| Recreational & social support / programs | Yes  No |  |  |  |  | Privately  Disability / injury insurer  Public health  Other |
| Medical care or nursing, pathology, imaging | Yes  No |  |  |  |  | Privately  Disability / injury insurer  Public health  Other |
| Other (e.g., pensions, allowances, assistive technology) Details: | Yes  No |  |  |  |  | Privately  Disability / injury insurer  Public health  Other |
